# Single patient in *GCK*-MODY family successfully re-diagnosed into *GCK*-PNDM through targeted next-generation sequencing technology

**DOI:** 10.1007/s00592-015-0786-0

**Published:** 2015-06-30

**Authors:** Karolina Antosik, Piotr Gnys, Elisa De Franco, Maciej Borowiec, Malgorzata Mysliwiec, Sian Ellard, Wojciech Mlynarski

**Affiliations:** Department of Clinical Genetics, Medical University of Lodz, Lodz, Poland; Department of Paediatrics, Oncology, Haematology and Diabetology, Medical University of Lodz, 36/50 Sporna Str., 91-738 Lodz, Poland; Department of Paediatrics, Diabetology and Endocrinology, Medical University of Gdansk, Gdańsk, Poland; Institute for Biomedical and Clinical Science, University of Exeter Medical School, Exeter, UK

To the Editor,

In light of recent findings concerning the next-generation sequencing (NGS) of our previously reported patient, we feel it necessary to briefly review our research and provide updated results.

In March 2011, we described the case of a female infant diagnosed with a heterozygous p.Gly223Ser mutation in the glucokinase gene (*GCK*) [1]. Heterozygous mutations of the *GCK* gene usually cause a mild clinical phenotype characterized by moderately elevated fasting hyperglycemia with slightly elevated levels of glycated hemoglobin, although the clinical course of diabetes may be highly variable. In contrast, homozygous or compound heterozygous mutations in this gene result in early onset of diabetes in the initial days of life, as well as pronounced hyperglycemia, ketoacidosis, and a severe clinical condition [2].

Although ten family members of the patient are also heterozygous carriers of the p.Gly223Ser mutation, she was the only one who did not present the heterozygous *GCK*-MODY phenotype. In fact, she exhibited severe hyperglycemia (765 mg/dl), dehydration, glucosuria, and ketoacidosis (pH 7.09, BE 14 mM) on the day after her birth. For the first 72 h after diagnosis, she was treated with 0.1–0.3 units/kg/h intravenous insulin for persistent hyperglycemia: the mean value from that period equaled 340 mg/dl.

During this time, her DNA was directly sequenced using Sanger’s method to identify homozygous or compound heterozygous mutations in the *GCK* gene using an ABI 3130 genetic analyser and DNA Sequencing Analysis Software (Applied Biosystems, Foster City, CA, USA). Sequencer software v4.1.4 (GeneCodes, Ann Arbor, MI, USA) was used for the comparative analysis of evaluated sequences. In addition, the use of Sanger’s sequencing and multiplex ligation-dependent probe amplification technique (MLPA) did not detect mutations or deletions in other known genes associated with monogenic diabetes. Only the heterozygous *GCK* p.Gly223Ser mutation was detected.

To further investigate the cause of such diversions from the expected phenotype, the DNA was reanalysed by next-generation sequencing in the reference laboratory in Exeter, UK. A targeted next-generation sequencing assay performed using an Illumina HiSeq 2000 sequencer (Illumina, San Diego, CA, USA) [3] indicated the presence of a second missense mutation in the *GCK* gene, c.1236A>G, which resulted in the amino acid substitution of glutamic acid to lysine at position 256 (p.Glu256Lys). This mutation was not visible in the results of the previous Sanger`s sequencing analysis and is now known to represent part of a compound heterozygous genotype resulting in PNDM (Permanent Neonatal Diabetes Mellitus). Reanalysis of the patient’s sample by Sanger’s sequencing revealed a rare single nucleotide polymorphism located in the DNA sequence covered by one primer used for PCR (rs573845006 reported in dbSNP build 142), which was *in cis* with the p.Glu256Lys mutation. This SNP resulted in allele dropout during PCR and previous misdiagnosis. Finally, a repeated Sanger’s sequencing of the *GCK* gene with redesigned primers also confirmed the p.Glu256Lys mutation (Fig. 1).

**Fig. 1:**
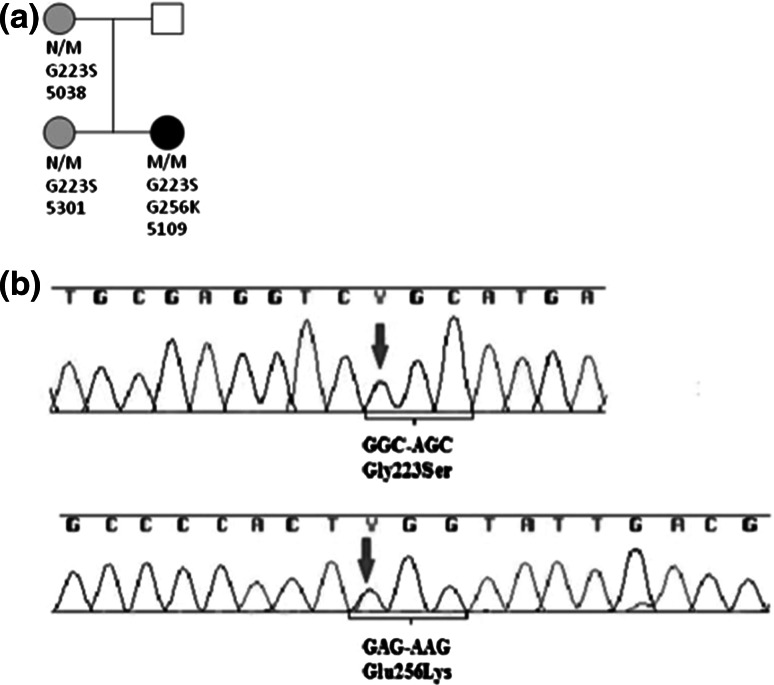
Genetic evaluation of the patient with GCK-PNDM originally previously reported [1]. **a** Family pedigree of the PNDM patient with compound heterozygous mutations in the *GCK* gene (No. 5109), *N* wild type, *M* mutation; **b** chromatograms obtained by direct sequencing according to Sanger, presenting a previously reported p.Gly223Ser mutation (*top* chromatogram) and a newly identified p.E256K *de novo* mutation (*bottom* chromatogram)

The p.Glu256Lys mutation was not inherited from the patient’s mother who is a carrier for the p.Glu223Ser mutation. We were not able to obtain biological material from the father of the patient. Since he did not report any symptoms of glucose metabolism disorders at the age of 38 years (fasting glucose 76 mg/dl; HbA1c 5.1 %; OGGT normal) and no family history of diabetes, and the p.Glu256Lys substitution is known in literature as causal for *GCK*-MODY phenotype, one may speculate that this mutation occurred as *de novo* in our PNDM patient. This is unusual finding leading to *GCK*-PNDM.

This case reinforces the need to remain aware of the potential for technology, reagents or other unforeseeable external factors to influence the results. Extreme caution is advised, both in diagnosing and in excluding some disorders, particularly in the case of such an extraordinary phenotype.

Approaching 10 years from the introduction of next-generation sequencing to widespread use, there is currently no doubt as to its usefulness [4]. Our laboratory is just one example of an institution, which has successfully incorporated NGS techniques into its daily workload, showing that new technologies may significantly improve the efficacy of tests, even those, which are retrospective in nature.

## References

[1] Borowiec M, Mysliwiec M, Fendler W, Antosik K, Brandt A, Malecki M, Mlynarski W (2011). Phenotype variability and neonatal diabetes in a large family with heterozygous mutation of the glucokinase gene. Acta Diabetol.

[2] Demirbilek H, Arya VB, Ozbek MN, Houghton J, Baran RT, Akar M, Tekes S, Tuzun H, Mackay DJG, Flanagan SE, Hattersley AT, Ellard S, Hussain K (2015). Clinical characteristics and molecular genetic analysis of 22 patients with neonatal diabetes from the South-Eastern region of Turkey: predominance of non-KATP channel mutations. Eur J Endocrinol.

[3] Ellard S, Lango Allen H, De Franco E, Flanagan SE, Hysenaj G, Colclough K, Houghton JAL, Shepherd M, Hattersley AT, Weedon MN, Caswell R (2013). Improved genetic testing for monogenic diabetes using targeted next-generation sequencing. Diabetologia.

[4] Van Dijk EL, Auger H, Jaszczyszyn Y, Thermes C (2014). Ten years of next-generation sequencing technology. Trends Genet.

